# The Quality of Life of Families of Children and Adolescents with Adolescent Idiopathic Scoliosis and the Adaptability and Cohesion of Families in the Patients’ Assessment

**DOI:** 10.3390/jcm15082816

**Published:** 2026-04-08

**Authors:** Barbara Cyran-Grzebyk, Gabriela Kołodziej-Lackorzyńska, Joanna Majewska, Daniel Szymczyk, Justyna Wyszyńska, Lidia Perenc

**Affiliations:** 1 Faculty of Health Sciences and Psychology, Collegium Medicum, University of Rzeszów, Kopisto Avenue 2a, 35-959 Rzeszów, Polanddszymczyk@ur.edu.pl (D.S.);; 2 Medical Faculty, Collegium Medicum, University of Rzeszów, Warzywna Avenue 1a, 35-310 Rzeszów, Poland

**Keywords:** family quality of life, juvenile idiopathic scoliosis, family adaptability, family cohesion, chronic disease

## Abstract

**Objectives:** Adolescent idiopathic scoliosis (AIS) may negatively affect both the quality of life of adolescents and the quality of life of their families (FQOL). Therefore, the analysis of objective and subjective determinants of FQOL in families of children and adolescents with AIS undergoing long-term conservative treatment becomes important and will allow for a better understanding of factors that may have a significant impact on the prognosis and clinical treatment outcomes. **Methods:** The analysis covered a total of 200 families of children and adolescents aged 7–18 from the Podkarpackie region (Poland). The medical history chart and the original physical examination card, as well as the Family Adaptability and Cohesion Scales (FACES III) and the Family Quality of Life Scale (FQOL), were used in this study. **Results:** Families of adolescents without AIS demonstrated significantly higher levels of family cohesion and adaptability compared with families of adolescents with AIS (*p* < 0.001). The mean overall FQOL score was significantly lower in the AIS group (75.33 ± 9.18) than in the control group (86.97 ± 7.91; *p* < 0.001, r_rb_ = 0.58). Multivariate analysis indicated that family adaptability was an independent predictor of FQOL in the AIS group, with higher adaptability associated with lower overall FQOL and reduced scores in parental functioning and emotional well-being domains. **Conclusions**: A long process of AIS treatment can cause crisis situations for patients and their families and influences both the physical and mental health of patients by changing their family’s quality of life (FQOL). Early identification of families characterized by diminished cohesion and adaptability enables the integration of psychopedagogical support and family consultations into standardized care. Such a multidimensional approach may enhance therapeutic prognosis and accelerate the rehabilitation process.

## 1. Introduction

Adolescent idiopathic scoliosis (AIS) is a very common spinal deformity in children and adolescents. In numerous publications, the authors state that the peak of its diagnosis and prevalence is during adolescence; meanwhile, according to the World Health Organization (WHO), it concerns “the stage of life between childhood and adulthood, at the age between 10 and 19 years of age”. This period is a time of intense physical, cognitive, and psychosocial development [1,2]. AIS is described as idiopathic because its etiology is yet unknown. Some theories regarding the etiology of AIS include hormonal causes, asymmetric growth, muscle imbalance, and genetic factors. AIS occurs in 0.47–5.2% of children and adolescents and more often in girls [3]. Scoliosis is accompanied by deformation of the spine in three planes; hence, medical intervention including conservative treatment (physiotherapy, bracing) or surgery is required. Lack of appropriate AIS treatment results in visible deformities within the spine itself and subsequently in the chest and pelvis. In the next stage of disease progression, there are disorders in the lower limbs (e.g., difference in length) and upper limbs (restricted mobility of the shoulder joint), as well as decreased cardiorespiratory efficiency and physical fitness disorders. Additionally, AIS also affects respiratory function, thus adding to the burden of disease for patients and their families [4]. There is also an aesthetic problem concerning the overall appearance of the adolescent patients. Progress and severity of the disease symptoms can result in a decrease in the quality of life (QOL) of people diagnosed with AIS and the quality of life of their families (FQOL), because visible deformities and a long process of conservative treatment cause stressful situations and psychological problems [5,6].

In addition, orthopedic supplies in the form of a scoliosis brace are sometimes unacceptable to young patients, which manifests itself in the form of reluctance, stress, aggression, and lack of cooperation with the parents and medical staff (doctor and physiotherapist). It is known and obvious that, in order for the AIS treatment to be effective, self-discipline is necessary, as well as the acceptance of various forms of therapy that are sometimes difficult to bear by adolescent patients. Hence, the people involved in this long-term treatment process, i.e., the doctor, the physiotherapist, and the parents, must work together [7].

For this reason, in clinical practice, in addition to the clinical and functional assessment of patients with AIS, the emotional state as well as the patient’s functioning in everyday life must be considered. The correlation between the social, emotional, and physical state and the severity of somatic symptoms has been observed for a very long time. For this reason, the assessment of the FQOL of patients treated conservatively for AIS and their functioning in everyday life seems to be very crucial and becomes an element that should be taken into account in the planning and implementation of the treatment process [8].

In this context, the family environment plays a crucial role in supporting adolescents throughout the therapeutic process. Parents and other family members often participate actively in treatment supervision, provide emotional support, and help adolescents adjust to lifestyle modifications required by therapy [9].

The family is the original and natural community in which a person grows up and fulfills their needs, i.e., love, reciprocity, durability and cooperation. As research shows, both in the USA and in liberal Sweden, the participation of both genders in the child’s educational process is absolutely crucial. The family system is based on and characterized primarily by cohesion, order, cyclical causation, hierarchical structure and adaptive self-organization [10]. Chronic disease directly affects not only the sick person, but also his relatives, causing a drastic change in the reality of the family in every aspect of family life and family functioning [9,11].

Due to the long duration of treatment, scoliosis can be classified as a chronic disease. Long-term treatment leads to crisis situations in the lives of patients and their relatives and changes their current, often established, values system, as well as the rhythm of life. Family members face coping with stress, anxiety, constant uncertainty, and new responsibilities. The family and the patient themselves face numerous challenges in terms of adaptability and cohesion to the new situation, which in turn affects the FQOL [12]. Cohesion is defined as the degree of emotionality and bonding between family members, while adaptability refers to the ability of the family system to change in response to situational and developmental stress, in this case, at the beginning of a long treatment, which sometimes ends in necessary surgical intervention [13]. Both the cohesion and adaptability of families seem to be an important and positive factor in the treatment of children with AIS [14]. It is worth emphasizing the important fact that, in families with children with special needs regarding healthcare, other interdisciplinary support concerning cohesion and adaptability can create opportunities for better family participation and improve the FQOL [15].

Therefore, the analysis of objective and subjective determinants of FQOL in families of children and adolescents with AIS undergoing long-term conservative treatment becomes important and will allow for a better understanding of factors that may have a significant impact on the process, prognosis, and effects of treatment.

## 2. Materials and Methods

### 2.1. Participants and Study Design

The analysis covered a total of 200 families of children and adolescents with an age range of 7 to 18 from the Podkarpackie Province (Poland). The study group consisted of 100 patients with AIS and their families and a control group of 100 peers without AIS and their families. In both groups, the female gender was predominant. In the control group, an attempt was made to replicate the gender structure of the study group.

The aim of the study was to compare families of children and adolescents with AIS with families of their peers without scoliosis. The study group consisted of 100 families of children and adolescents aged 9–18 years with a diagnosis of AIS confirmed radiologically (Cobb angle ≥ 10°), who were actively undergoing conservative treatment, including physiotherapy using the proprioceptive neuromuscular facilitation (PNF) method. The control group was matched to the study group in terms of sample size, age, sex, and family structure and included 100 families of children and adolescents without scoliosis or other chronic diseases or developmental disorders. Only children from intact families were eligible for inclusion. Written informed consent was obtained from both parents and participating children prior to enrollment. The sampling procedure was non-random. This cross-sectional study was conducted from January 2019 to December 2020 following institutional approval and a positive opinion from the Bioethics Committee of the University of Rzeszów (resolution No. 4/11/2018 on 8 November 2018). The selection of the sample was carried out in a non-random manner; all available participants meeting the eligibility criteria were qualified (recognition sample) [16]. Finally, the study group consisted of 100 patients aged 9 to 18, participating in rehabilitation treatment at the Clinical Rehabilitation and Education Center at the Provincial Clinical Hospital No. 2 in Rzeszow (Poland), due to their AIS. The control group was selected to maintain the same size, gender, and age structure as in the study group. Control participants were recruited from local schools and community settings in the same region and were matched to the AIS group in terms of age, sex, and family structure.

The following research tools were used: the author’s original medical history and physical examination card created for the purpose of the study, the Family Adaptability and Cohesion Scales (FACES III), and the Family Quality of Life Scale (FQOL). The study was conducted once. All participants were informed about the procedures of this study, and written informed consent was obtained from all subjects before participating in this study.

### 2.2. Group Characteristics

Girls with AIS constituted 83% of the entire study group versus 17% of boys, while girls without AIS constituted 78% of the control group and boys 22%. There were no statistically significant differences between the study and control groups. The mean age of children and adolescents from both groups was 14. In both groups, children and adolescents were of similar age and raised in full families (single-parent family was one of the criteria excluding them from the study). The living environment was divided into urban and rural. A total of 74% of families from the study group came from an urban environment, while 26% of families of children and adolescents with AIS came from a rural environment. In the control group, 86% of families of children and adolescents without AIS came from an urban environment and 14% from a rural environment (Table 1).

### 2.3. Procedures

#### 2.3.1. Family Adaptability and Cohesion Scales (FACES III)

The first version of the FACES scale was developed by Portner and Bell in 1978. The construction of the scale was based on assumptions taken from the systemic concept of the family developed by Olson and colleagues from the University of Minnesota. The purpose of the scale is to measure the 2 basic dimensions included in this model: family adaptability and cohesion [17].

The FACES III questionnaire consists of 20 items, 10 of which correspond with each of the dimensions of the functioning of the family system (10 adaptability, 10 cohesion). The advantage of this scale is the high correlation between individual items within a given sub-scale and its overall score, as well as ease of application. After reading the questions contained in the questionnaire, all respondents from both groups (family members: child, mother, and father) provided answers separately on a 5-point rating scale (from 1—almost never to 5—almost always). They determined how often a certain type of behavior occurred in their family. Scores for both sub-scales were calculated separately, with higher scores indicating stronger levels of these dimensions [18,19].

#### 2.3.2. The Family Quality of Life Scale (FQOL)

The FQOL questionnaire was used to assess the level of quality of life related to the health of the family and its individual members. The scale is used to assess family members’ perceptions of their satisfaction with various aspects of the family’s quality of life and includes five sub-scales: family interaction, parenting, emotional well-being, physical and material well-being, and support for people with disabilities. Respondents answered the questions according to standardized 5-point categories: very dissatisfied, dissatisfied, hard to say, satisfied, and very satisfied. The scale questionnaire was completed by the child and parents (father, mother) individually. If the general and sub-indices of the FQOL questionnaire are positively oriented, it means that they have a high score, which indicates a high level of FQOL. The questionnaire was based on the principle of providing answers from the perspective of the previous 12 months. The FQOL questionnaire was completed by all family members from both the study and control groups [20]. Higher scores reflect higher levels of perceived family quality of life. Family quality of life was assessed using the FQOL developed by Brown et al. The instrument has been used in studies conducted in Polish populations, including research evaluating family quality of life among families with a member with an intellectual disability [21].

### 2.4. Statistical Analysis

The statistical analysis of the obtained results was performed using the R software (R Foundation for Statistical Computing, Vienna, Austria), version 3.6.2 [20]. Quantitative features in both groups were described by analyzing quantitative variables (expressed in numbers) and calculating the mean (M), standard deviation (SD), median, quartiles, minimum, and maximum. The analysis of qualitative variables (not expressed in numbers) was carried out by calculating the number and percentage of occurrences of each of the specified values. The comparison of the values of qualitative variables in groups was made using the chi-square test (with Yates’ correction for 2 × 2 tables) or Fisher’s exact test when low expected numbers appeared in the tables. The comparison of the values of quantitative variables in two groups was performed using the Mann–Whitney test (*p*), and the comparison of the values of quantitative variables in three or more groups was performed using the Kruskal–Wallis test. If statistically significant differences were found, a post hoc analysis was performed using Dunn’s test to identify the groups in which these statistically significant differences occurred. Correlations between quantitative variables were calculated using Spearman’s rank correlation coefficient (R). Due to the lack of normal distribution of statistical data, the strength of the association between the analyzed variables was assessed using the rank–biserial correlation coefficient (r_rb_). The magnitude of the obtained associations was interpreted according to conventional criteria proposed by J. Cohen. According to this classification, coefficient values of r_rb_ ≥ 0.1 were interpreted as indicating a weak effect, r_rb_ ≥ 0.3 as a moderate effect, and r_rb_ ≥ 0.5 as a strong effect [22,23].

Multivariate analysis of the independent influence of many variables on the quantitative variable was performed using the linear regression method. The results are presented as regression model parameter values with a 95% confidence interval. For the purpose of the analysis of results, the level of statistical significance was assumed at *p* < 0.05. All *p* values below 0.05 were interpreted as statistically significant (*p* < 0.05).

## 3. Results

### 3.1. Comparison of Family Cohesion and Adaptability in the Study and Control Groups

The following results are based on self-reports provided by children and adolescents from both the study and control groups. Based on the comparative analysis of the results of the FACES III scale, presented in Table 2, completed by children and adolescents with AIS and peers from the control group, it was shown that the dimensions of family cohesion (*p* < 0.001) and adaptability (*p* < 0.001) were significantly higher in the assessment of children and adolescents from the control group. These findings indicate statistically significant differences in the perception of family functioning between the two groups.

### 3.2. The Level of Family Quality of Life for Children and Adolescents with AIS Versus the Level of Family Quality of Life for Children and Adolescents from the Control Group

The analyses presented below refer to responses provided by children and adolescents who completed the FACES III and FQOL questionnaires.

Statistically significant differences in the FQOL level in all analyzed areas of the scale, based on the assessments of children and adolescents with AIS and children and adolescents from the control group, are presented in Table 3. The FQOL level assessed by children and adolescents from the control group was significantly higher in all FQOL sub-scales: family interactions, performing parental functions, emotional well-being, physical/material well-being, and problems with disabilities. The *p* values for all the FQOL sub-scales were below *p* < 0.001. The results presented in Table 3 refer to assessments provided by children and adolescents from both groups. Children and adolescents from the control group reported significantly higher scores in all analyzed FQOL domains compared with participants from the AIS group (*p* < 0.001). These findings demonstrate statistically significant differences in perceived family quality of life between the study and control groups. The analysis of rank–biserial correlation coefficients revealed a statistically significant asymmetry in the distributions in favor of the control group across all analyzed dimensions of FQOL. The strongest association effects were observed for general family quality of life (r_rb_ = 0.58) and disability-related problems (r_rb_ = 0.49). The magnitude of the obtained parameters identified the analyzed variables as key determinants differentiating the study group from the control group.

### 3.3. The Level of Family Quality of Life and Its Cohesion and Adaptability in the Assessment of Children and Adolescents with Idiopathic Scoliosis

The results presented in this subsection are based on self-reports provided by children and adolescents from both the study and control groups. There was no significant correlation between family cohesion (FACES III) assessed by children and adolescents with idiopathic scoliosis and the level of FQOL. The results presented in Table 4 show that none of the Family Quality of Life sub-scale results had any significant impact on its level. In the group of patients with idiopathic scoliosis, the impact of the disease and the need for its long-term treatment did not reveal a significant correlation between family cohesion and the level of Family Quality of Life. Statistical analysis conducted in the study group showed that the adaptability of the family (FACES III) correlated significantly and negatively with some of the FQOL results: overall FQOL (R = −0.231, *p* = 0.021), parenting (R = −0.248, *p* = 0.013), and emotional well-being (R = −0.31, *p* = 0.002). The demonstrated correlation shows that the higher the level of family adaptability, the lower the FQOL level in the above-mentioned areas in the assessment of children and adolescents with AIS. In the AIS group, statistically significant negative correlations were observed between family adaptability (FACES III) and selected FQOL domains, including overall FQOL (R = −0.231, *p* = 0.021), parenting (R = −0.248, *p* = 0.013), and emotional well-being (R = −0.31, *p* = 0.002). These results indicate that higher adaptability scores were associated with lower scores in these FQOL domains in the assessments provided by children and adolescents with AIS (Table 4).

### 3.4. Identification of Positive and Negative Predictors of the Family’s Quality of Life Level for Children and Adolescents from the Study Group Based on Multivariate Analysis

The following multivariate analyses are based on self-reports provided by children and adolescents from the study group. The multivariate analysis carried out in the study group showed significant correlations between family cohesion and adaptability with the level of FQOL in most of its areas (Table 5). Based on the obtained results of the linear regression model, it was found that an important, independent predictor of overall quality of life, emotional well-being, and problems with disability (FQOL) in the assessment of children and adolescents from the study group was family adaptability (FACES III) (*p* ˂ 0.007). Subsequently, the regression parameter for family adaptability was −0.384, which means that a 1-point higher level of adaptability lowered the level of overall quality of life by an average of 0.384 points. Similarly, for family adaptability (FACES III), the regression parameter in the area of disability problems (FQOL) was −0.13, which means that an additional point obtained in the assessment using the FACES III scale (higher dimension of adaptability) reduced the FQOL level in the area of disability problems by an average of 0.130 points (Table 5). Multivariate regression analysis showed that family adaptability (FACES III) was significantly associated with several FQOL domains assessed by children and adolescents in the study group, including overall quality of life, emotional well-being, and problems related to disability (*p* < 0.007). The regression coefficient indicated that higher adaptability scores were associated with lower scores in these FQOL domains.

Based on the linear regression model, it was also shown that family adaptability was a statistically significant and independent predictor of the FQOL level in the area of parental function and emotional well-being in the assessment of children and adolescents from the control group—*p* < 0.016 and *p* < 0.002, respectively (Table 5). Family adaptability (FACES III) in two out of five FQOL areas was considered a positive predictor. In the remaining areas of the FQOL level, both cohesion and adaptability (FACS III) were not statistically significant in the assessment of children and adolescents without scoliosis.

### 3.5. The Level of Quality of Life of Families and Their Cohesion and Adaptability in the Assessment of Children and Adolescents from the Control Group

The results presented in Table 6 refer to the assessment of the relationship between family cohesion (FACES III) indicated by children and adolescents from the control group and the level of FQOL. The analysis showed that family cohesion was only significantly and positively correlated with disability problems (R = 0.234, *p* = 0.019). Statistical analysis showed no significant relationship between family adaptability (FACES III) and FQOL level (Table 6). Compared to cohesion, non-AIS children and adolescents did not notice the importance of family adaptability on FQOL in any of the five areas. This result may indicate that family cohesion is more important than adaptability in their subjective assessment.

## 4. Discussion

As part of the AIS treatment process, the priority goal of conservative management is to stop or decrease the progression of spine deformity, although an important goal of therapy is also to improve the quality of life of patients and their families. In recent years quality of life (QOL) assessment has become one of the most important issues in modern science and is the subject of research in many scientific fields, including medicine and health sciences [24,25]. Already, at the beginning of the 1990s, Schipper introduced the concept of Health-Related Quality of Life (HRQOL) to modern medicine. He emphasized the fact that HRQOL is the effect of the disease and its treatment, subjectively or objectively perceived by the patient themself [26]. Thus, considered to be dependent on the state of human health and determined by his overall condition, QOL allowed us to assess the factors influencing it in a way that was directly related to health. It has become a prerequisite to take into account human functional capabilities, including the ability to actively fulfill the needs of everyday life, participate in social life, perform or assume social roles, and maintain emotional and intellectual capacity and economic position [27,28]. Psychosocial studies on the population of children and adolescents with scoliosis allow us to identify difficulties such as problems with self-esteem, body image, fear of peer evaluation, and even depression. In turn, a positive attitude, social support and good self-esteem can contribute to better patient cooperation in the treatment and rehabilitation process.

The present study examined the relationship between family cohesion, adaptability, and FQOL in families of adolescents with AIS undergoing conservative treatment. The results showed that families of adolescents with AIS reported significantly lower levels of family cohesion and adaptability compared with families of adolescents without scoliosis. In addition, adolescents with AIS reported lower levels of FQOL across all analyzed domains, suggesting that the long-term treatment process associated with AIS may affect family functioning and perceived quality of life.

The time of illness and its treatment is difficult for the whole family and is a challenge to parents that can lead to significant changes in the lifestyle and maintenance of the entire family system. The perspective of deformation of the body of a child during adolescence and youth and the important role of time that must be taken into account for the treatment to be effective causes stress for all people involved in the treatment process. Expectations on various levels of family functioning and mutual expectations between its individual members intensify during this period. It seems reasonable to use the term disability in the AIS-patient context, because for parents of a child with AIS, it can cause such an effect [29,30]. Multivariate analyses revealed different patterns of associations between family functioning and FQOL in the two groups. In the AIS group, family adaptability emerged as a significant predictor of several FQOL domains; however, higher adaptability was associated with lower levels of overall FQOL and selected subdomains. This unexpected direction should be interpreted cautiously and may reflect ongoing attempts by families to reorganize and cope with the demands of long-term treatment rather than indicating optimal family functioning.

In the literature on the subject, one can find numerous articles on the QOL of people with AIS, but this problem is rarely presented and analyzed in the context of the whole family. No previous studies have investigated the relations between FQOL and FACES III in patients with AIS [31,32]. It is obvious and undisputed that the family is a very important social unit that is deeply involved in the daily care of a child with AIS. The family encounters a number of problems, including the possible decrease in their QOL and the need to adapt to the new situation and maintain cohesion. Illness and disability of any family member, including a child, disturbs the family system’s functioning. Kahanovitz and Weiser studied 72 adolescents with AIS aged 12–16 and proved that patients from single-parent families generally have lower levels of FQOL. At the same time, it turned out that the attitudes of mothers to the child’s disease and abnormal body shape were more favorable than that of fathers, which improved the QOL. Researchers have emphasized the fact that the mental state of parents directly affects children; therefore, the relationship between the parent and the child as an adolescent patient should be considered during treatment [33].

Since previous studies assessing similar correlations in such a group of patients are not available, this research was based on results of similar studies conducted on a group of patients with other chronic diseases.

Zhou et al. studied 55 cancer patients. Statistical analysis of the results showed that adaptability and family cohesion indirectly affected the presence of suicidal thoughts, in connection with coping mechanisms and depression. Adaptability and cohesion of the family turned out to be protective factors against suicidal thoughts in cancer patients. Improving the family support system and coping mechanisms can be used to help cancer patients [34]. On the other hand, Zhang et al., in a study evaluating 61 patients with chronic bipolar disease, observed that higher cohesion and adaptability levels in their families slightly increased the tendency to improve the treatment process of patients. Family cohesion, adaptability, and functioning were interrelated. The family system and family functioning, in their opinion, are important factors that clinicians should consider while treating people with bipolar disease [35]. In our own research, family cohesion and adaptability, as assessed by adolescents from both groups, were significantly more pronounced in families of children and adolescents without AIS in comparison to families of children and adolescents with idiopathic scoliosis. Therefore, therapy focused on cohesion and adaptability in families of children and adolescents with AIS should be considered. It is worth mentioning that the assessment and analysis of family cohesion and adaptability in the group of families of children and adolescents with AIS and in the control group showed a higher level of family adaptability for children and adolescents with AIS, but this resulted in a decrease in the level of general FQOL (R = −0.231, *p* = 0.021) and FQOL in the areas of performing parental function (R = −0.248, *p* = 0.013) and emotional well-being (R = −0.31, *p* = 0.0 02). Lei and Kantor were assessing FQOL, family cohesion, and adaptability in a group of Chinese families of children diagnosed with autism. A total of 163 children’s parents and guardians participated in the study. The authors of the publication assessed the role of family cohesion and adaptability in the relationship between social support and FQOL. Researchers found that social support had a positive effect on FQOL, and family cohesion and adaptability were part of the relationship between social support and caregiver satisfaction assessed with FQOL, which was also confirmed by our own research [36]. Significant correlations between FACES III and FQOL were found. The results showed that, for children and adolescents with AIS, higher adaptability of the family resulted in a decrease in the level of general FQOL and FQOL in the areas of parenting and emotional well-being. However, in the opinion of children and adolescents without AIS, a higher level of family cohesion increases the level of FQOL in the area of problems with disability only.

Interesting results considering the assessment of FQOL and FACES III were published by Rosalini et al. The authors assessed the relationship between QOL, family cohesion, and the sociodemographic factors of the families. This cross-sectional study, conducted on 385 respondents, showed that independent variables such as the sociodemographic factor, self-reported health status, family cohesion, and adaptability (FACES III) significantly affected the dependent variable, which was the QOL in this study. According to the study results, the best QOL was associated with ages up to 36 years (R = 2.15), tertiary education (R = 1.54), good/very good health (R = 6.39), no current health problems (R = 5.68), and a high level of family cohesion and moderate adaptability (R = 2.23). People from families with moderate and high family cohesion more often obtained better FQOL results than people from families with lower level of cohesion [37]. This was also confirmed in our own research, which showed that family adaptability was a predictor of a higher FQOL level, especially in the area of parenting functions in adolescents without AIS. However, for children and adolescents with AIS, a higher level of family adaptability resulted in a decrease in the level of general FQOL (R = −0.231, *p* = 0.021) and FQOL in the areas of parenting functions (R = −0.248, *p* = 0.013) and emotional well-being (R = −0.31, *p* = 0.002). Kim and Yoo evaluated 74 children aged 10–15 diagnosed with cancer. The aim of their work was to examine the correlation of FACES III patient results with their peer group and their teachers, which led to an important conclusion. Adaptability and family cohesion (*p* = 0.535, *p* < 0.001) and relationships with friends (R = 0.520, *p* < 0.001) and teachers (R = 0.318, *p* < 0.01) were significantly associated with cohesion. The authors of the study call for support for parents of chronically ill children in order to improve their functioning. They also recommend developing special support and educational programs for parents to promote family adaptation and cohesion and educational programs for colleagues and teachers [38]. Similarly, studies on the QOL of families of children with spina bifida confirm the need to improve their adaptability and cohesion. This fact is becoming more and more important in family-oriented support programs in order to provide them with the best possible support in the process of treatment and the improvement of QOL [39]. To the best knowledge of the authors of this study, results of our research are the first analysis of the correlation between FACES III and the level of FQOL among families of children and adolescents with AIS. Previous studies indicate that chronic illness in children and adolescents can substantially affect family functioning and psychosocial well-being. Long-term treatment and increased caregiving responsibilities may place additional emotional and organizational demands on families, influencing both family dynamics and perceived quality of life. Although numerous studies have examined quality of life in adolescents with AIS, relatively few have explored these relationships from the perspective of the family system.

Lewandowska-Walter et al. researched 33 patients with Marfan syndrome and those without chronic diseases. They showed that people with Marfan syndrome perceived their families as having a lower level of cohesion and adaptability and lower life satisfaction level, as measured by the Satisfaction with Life Scale (SWLS), compared to the control group [40]. The significance of the FQOL correlation with FACES III is discussed in the article by Yee et al. The researchers examined the adaptability and cohesion of families, including mothers and patients treated for mental disorders. The results showed that adolescents with mental disorders related to psychosis and their mothers reported lower adaptability and family cohesion than their healthy peers [9]. Our own research shows that the FQOL level of children and adolescents with AIS was statistically significantly lower than the FQOL level of children and adolescents without AIS in the assessment of both adolescents and their parents in general QOL and in all FQOL sub-scales. It is worth emphasizing that family adaptability turned out to be a negative predictor of the level of FQOL in the area of general QOL and emotional well-being, as confirmed by the results of the multivariate analysis on the study group. On the other hand, cohesion and adaptability turned out to be a positive predictor in their assessment in the area of family interactions and disability problems. The purpose of the article by Barłog et al. was to assess the sense of loneliness of a family with a disabled child and its level of cohesion and adaptability in the time of the SARS-CoV-2 Coronavirus pandemic. De Jung Gierveld scale and the FACES III family questionnaire were used to evaluate the sense of loneliness. After examining 131 people and parents of children with intellectual disabilities, they showed a correlation between the sense of loneliness and the level of family cohesion and adaptability. The presented results indicate a strong relationship between the cohesion and adaptability of their families, including their individual members, with the level of loneliness. Along with the increase in isolation and loneliness and the need to care for a sick child, the level of adaptability to the prevailing situation and family cohesion were decreased [41].

The review of contemporary literature considering the use of the FACES III scale and its correlation with the FQOL level of patients with AIS conducted by the authors of this study indicates the difficulty of comparing them to the results of our own research. This is due to the fact that other researchers do not investigate similar correlations, such as the impact of family cohesion and adaptability on the FQOL level, evaluating the impact of AIS itself on the patient’s QOL only.

There are some limitations observed in our study. The inclusion criteria in the group of children and adolescents with idiopathic scoliosis in the study was a current X-ray of the entire spine (it was clearly specified that it should not have been older than 6 months). However, there was a large group of children and adolescents with AIS who had X-rays taken only once a year, as was recommended by the attending physician, and they were excluded from the study group. If we had included those patients in the study, the group would have been much larger. Additionally, children and adolescents with a history of surgical intervention for AIS were not included in the research.

The study had a cross-sectional design, which precludes drawing conclusions about cause-and-effect relationships. It is not possible to determine which variable constitutes the cause and which the effect. It is only possible to observe the co-occurrence of phenomena. Cross-sectional studies also do not allow for the tracking of analyzed phenomena over time.

It should also be acknowledged that potential, unmeasured confounding factors may have been present, which could lead to results that do not accurately reflect the family’s actual capacity for adaptation and cohesion but rather represent its struggle with internal or external difficulties. Such factors may include the Cobb angle value, the duration of brace treatment, low socioeconomic status, mental health problems in one or both parents, as well as stressful life events, or the level of social support. These findings suggest that psychosocial interventions may play an important role in supporting patients undergoing conservative treatment for AIS.

Another limitation of the study is that the characterization and comparison of the study and control groups did not take into account factors such as the number of children and the economic status of the families. A more detailed analysis of additional factors that may influence FQOL and FACES III scores should be considered in future research.

The limitations of using FACES III also include its reliance on self-report, which is inherently subjective. Responses may be influenced by the respondent’s current mood, general well-being, social desirability, or even the desire to present oneself in a more favorable light, as well as by low self-esteem. Self-assessment may also vary considerably over time. This lack of stability in evaluation over the long term may complicate the analysis and comparison of results. Furthermore, common interpretations of FACES III suggest that ideal families fall within the mid-range of adaptability and cohesion; however, extremes (very high and very low adaptability and cohesion) may be equally adaptive in certain contexts or for certain stages of family development, which the instrument does not account for. This can lead to a misunderstanding of the complexities of family dynamics.

However, the results of our study presented in this paper suggest and encourage further research, including multi-center and international studies, to better understand the role of family cohesion, adaptability and FQOL in the process of diagnosing, planning, and treating AIS patients.

## 5. Conclusions

Families of children and adolescents without AIS demonstrated significantly higher levels of cohesion and adaptability compared with families of adolescents with AIS. Family adaptability was identified as a significant factor differentiating Family Quality of Life (FQOL) in both groups. In the AIS group, higher adaptability was associated with lower overall FQOL and reduced FQOL in the domains of parental functioning and emotional well-being. Multivariate analysis further showed that family cohesion acted as a positive predictor of FQOL in the domains of family interactions and physical/material well-being, whereas adaptability functioned as a negative predictor in the domain of disability-related problems. In contrast, in the control group, adaptability emerged as a positive predictor of FQOL in parental functioning and emotional well-being, while family cohesion did not demonstrate a statistically significant association with FQOL.

The large effect size observed for overall family quality of life indicates a marked polarization in the well-being of the studied families and highlights the relevance of a systemic framework in the assessment of family functioning. From a clinical perspective, these findings support the integration of psychoeducational interventions, psychological counseling, and programs aimed at strengthening family adaptation resources into routine healthcare practice. Such approaches may contribute to improved functioning of the family system and enhanced therapeutic effectiveness.

The moderate effect size observed for disability-related problems suggests that functional and structural barriers constitute a significant factor undermining the quality of life and daily functioning of the family system. This finding underscores the necessity of expanding therapeutic education and providing targeted support for home environment adaptation.

## Figures and Tables

**Table 1:** Characteristics and comparison of the study and control group.

	Study Group (n = 100)			Control Group (n = 100)			*p*
Parameter	Mean ± SD	Median Value	Quartile	Mean ± SD	Median	Quartile	
Age of children/adolescents (years)	13.83 ± 1.92	13.83 ± 1.92	12–16	14.09 ± 2.16	14	12–16	0.411
Age of mothers (years)	42.71 ± 4.71	42.71 ± 4.71	39–46	42.17 ± 5.04	41	39–45	0.589
Age of fathers (years)	43.56 ± 5.22	43.56 ± 5.22	40–47.25	43.69 ± 4.45	44	40–46.25	0.731
	n; %			n; %			
Gender							
Male		17; 17.00		22; 22.00			0.475
Female		83; 83.00		78; 78.00			
Living environment							
Village		26; 26.00		14; 14.00			0.052
City		74; 74.00		86; 86.00			
Structure of the family							
Intact family		100; 100.00		100; 100.00			1
Single-parent family		0; 0.00		0; 0.00			

n—sample size; mean ± SD—mean value and standard deviation; *p*—the Mann–Whitney test result (for quantitative variables), the chi-square test, or Fisher’s exact test result (for qualitative variables); and *p* < 0.05 indicates a statistically significant difference.

**Table 2:** Family cohesion and adaptability (FACES III) assessed by children and adolescents from the study and control groups.

	Study Group (n = 100)			Control Group (n = 100)			*p*	Z	r_rb_
Family Cohesion and Adaptability (FACES III)	Mean ± SD	Median	Quartile	Mean ± SD	Median	Quartile			
Family cohesion	23.48 ± 5.12	23	20–28	27.85 ± 7.12	27.50	23–34	<0.001 *	4.15	0.29
Family adaptability	27.38 ± 6.36	28	23–32	31.13 ± 5.79	29.50	27–36	<0.001 *	3.73	0.26

n—sample size; mean ± SD—mean value and standard deviation; Z—score; *p*—the Mann–Whitney test result; *—*p* < 0.05 indicates a statistically significant difference; and r_rb_—rank–bilateral correlation.

**Table 3:** FQOL level in families of children and adolescents with ASI and FQOL level in families of children and adolescents from the control group.

	Study Group (n = 100)			Control Group (n = 100)			*p*	Z	r_rb_
Family Quality of Life Level	Mean ± SD	Median	Quartile	Mean ± SD	Mediana	Quartile			
General family quality of life	75.33 ± 9.18	76	70–81	86.97 ± 7.91	88.5	81–93	<0.001 *	8.27	0.58
Family interactions	18.08 ± 4.85	18	16–19	20.85 ± 3.36	21	19–23.25	<0.001 *	6.18	0.44
Parental functions	18.3 ± 2.99	18	15.75–21	20.58 ± 2.98	21	19–22	<0.001 *	5.09	0.36
Emotional well-being	12.14 ± 2.44	12	10–14	13.81 ± 2.39	14	12–15	<0.001 *	4.40	0.31
Physical/material well-being	15.3 ± 2.62	15	13–17	17.71 ± 2.36	18	16–19	<0.001 *	6.09	0.43
Problems with disabilities	11.28 ± 2.81	11.50	9–13	14.09 ± 1.99	14	12–15	<0.001 *	6.96	0.49

n—sample size; mean ± SD—mean value and standard deviation; Z—score; *p*—the Mann-Whitney test result; *—*p* < 0.05 indicates a statistically significant difference; and r_rb_—rank–bilateral correlation.

**Table 4:** Correlation between family cohesion and adaptability (FACES III) and FQOL level in the assessment of children and adolescents with AIS.

	Family Cohesion (FACES III)			Family Adaptability (FACES III)	
Family Quality of Life	R	*p*	Family Quality of Life	R	*p*
Parental functions	0.121	0.231	Family interactions	0.045	0.659
Problems with disabilities	0.117	0.244	Physical/material well-being	0.03	0.766
General QOL	0.101	0.319	Problems with disabilities	−0.124	0.219
Emotional well-being	−0.019	0.854	General QOL	−0.231	0.021 *
Physical/material well-being	−0.032	0.751	Parental functions	−0.248	0.013 *
Family interactions	−0.11	0.275	Emotional well-being	−0.31	0.002 *

R—Spearman’s rank correlation coefficient value; *—*p* < 0.05 indicates a statistically significant correlation.

**Table 5:** Multivariate analysis of the influence of independent variables on individual FQOL levels assessed by children and adolescents with and without AIS.

Multivariate Analysis of the Influence of Independent Variables on Individual FQOL Levels Assessed by Children and Adolescents from Study Group					Multivariate Analysis of the Influence of Independent Variables on General FQOL Assessed by Children and Adolescents from Control Group				
Feature	Parameter	95% CI		*p*	Feature	Parameter	95% CI		*p*
Family cohesion (FACES III)	0.067	−0.316	0.451	0.731	Family cohesion (FACES III)	0.25	−0.079	0.579	0.14
Family adaptability (FACS III)	−0.384	−0.678	−0.089	0.013 *	Family adaptability (FACES III)	0.363	−0.019	0.745	0.066
Multivariate analysis of the influence of independent variables on family interactions assessed by children and adolescents from study group					Multivariate analysis of the influence of independent variables on family interactions (FQOL) assessed by children and adolescents from control group				
Family cohesion (FACES III)	−0.238	−0.438	−0.038	0.022 *	Family cohesion (FACES III)	0.098	−0.04	0.236	0.169
Family adaptability (FACES III)	0.109	−0.045	0.262	0.17	Family adaptability (FACES III)	0.049	−0.112	0.209	0.554
Multivariate analysis of the influence of independent variables on parental functions assessed by children and adolescents from study group					Multivariate analysis of the influence of independent variables on parental functions FQOL assessed by children and adolescents from control group				
Family cohesion (FACES III)	0.018	−0.109	0.145	0.784	Family cohesion (FACES III)	−0.013	−0.142	0.116	0.842
Family adaptability (FACES III)	−0.083	−0.18	0.015	0.101	Family adaptability (FACES III)	0.187	0.037	0.337	0.016 *
Multivariate analysis of the influence of independent variables on emotional well-being assessed by children and adolescents from study group					Multivariate analysis of the influence of independent variables on emotional well-being (FQOL) assessed by children and adolescents from control group				
Family cohesion (FACES III)	−0.033	−0.136	0.071	0.54	Family cohesion (FACES III)	0.084	−0.014	0.183	0.097
Family adaptability (FACES III)	−0.112	−0.192	−0.033	0.007 *	Family adaptability (FACES III)	0.183	0.068	0.297	0.002 *
Multivariate analysis of the influence of independent variables on individual on physical/material well-being assessed by children from study group					Multivariate analysis of the influence of independent variables on physical/material well-being (FQOL) assessed by children and adolescents from control group				
Family cohesion (FACES III)	0.053	−0.064	0.17	0.373	Family cohesion (FACES III)	0.078	−0.029	0.186	0.157
Family adaptability (FACES III)	−0.001	−0.091	0.089	0.98	Family adaptability (FACES III)	−0.023	−0.148	0.102	0.721
Multivariate analysis of the influence of independent variables on problems with disabilities assessed by children and adolescents from study group					Multivariate analysis of the influence of independent variables on problems with disabilities (FQOL) assessed by children and adolescents from control group				
Family cohesion (FACES III)	0.087	−0.032	0.207	0.154	Family cohesion (FACES III)	0.033	−0.052	0.117	0.449
Family adaptability (FACES III)	−0.13	−0.221	−0.038	0.007 *	Family adaptability (FACES III)	−0.075	−0.173	0.024	0.14

Multivariate linear regression: *p*—statistical significance coefficient; 95% CI—95% confidence interval; and *—*p* < 0.05 indicates a statistically significant relationship.

**Table 6:** Correlation between family cohesion and adaptability (FACES III) and FQOL level in the assessment of children and adolescents from the control group.

	Family Cohesion (FACES III)			Family Adaptability (FACES III)	
FQOL Level	R	*p*	FQOL Level	R	*p*
Problems with disability	0.234	0.019 *	Emotional well-being	0.176	0.08
Emotional well-being	0.177	0.077	Family interactions	0.059	0.563
Family interactions	0.121	0.232	Parental functions	0.053	0.602
General quality of life	0.096	0.344	Problems with disability	0.04	0.694
Physical/material well-being	0.005	0.964	General quality of life	0.039	0.702
Parental functions	−0.063	0.533	Physical/material well-being	−0.092	0.365

R—Spearman’s rank correlation coefficient value; *—*p* < 0.05 indicates a statistically significant correlation.

## Data Availability

The raw data supporting the conclusions of this article will be made available by the authors on request.
